# Cinnamic acid alleviates hypertensive left ventricular hypertrophy by antagonizing the vasopressor activity and the pro-cardiac hypertrophic signaling of angiotensin II

**DOI:** 10.3389/fphar.2025.1555991

**Published:** 2025-02-14

**Authors:** Yimeng Cui, Yawei Yang, Xinmiao Tang, Peiwei Wang, Jingang Cui, Yu Chen, Teng Zhang

**Affiliations:** ^1^ Yueyang Hospital of Integrated Traditional Chinese and Western Medicine, Shanghai University of Traditional Chinese Medicine, Shanghai, China; ^2^ Clinical Research Institute of Integrative Medicine, Shanghai Academy of Traditional Chinese Medicine, Shanghai, China; ^3^ Laboratory of Clinical and Molecular Pharmacology, Yueyang Hospital of Integrated Traditional Chinese and Western Medicine, Shanghai University of Traditional Chinese Medicine, Shanghai, China

**Keywords:** cinnamic acid, hypertension, left ventricular hypertrophy, cardiomyocytes, angiotensin II, pro-hypertrophic responses

## Abstract

**Background:**

Hypertension is the most common cause of pathological left ventricular hypertrophy, a condition causally associated with debilitating heart failure and cardiovascular events in hypertensive patients. It is well recognized that the disease burden of hypertension-linked heart failure remains unabated with existing treatments. New therapies controlling hypertensive left ventricular hypertrophy are thus required to decelerate or prevent the development of heart failure. Our previous study has demonstrated that cinnamic acid, a naturally occurring monocarboxylic acid, mitigates transverse aortic constriction-induced pressure overload-mediated cardiac hypertrophy. However, whether cinnamic acid is effective at controlling hypertensive left ventricular hypertrophy remains unknown. Angiotensin II (ang II) plays a pivotal role in driving the pathogenesis of hypertensive left ventricular hypertrophy. The current work thus investigates the therapeutic potential and pharmacological mechanisms of cinnamic acid in the context of ang II-mediated hypertensive left ventricular hypertrophy.

**Methods:**

Ang II-infused mice and cardiomyocytes were analyzed by histological, immunohistochemical, cellular and molecular biological methods to delineate the impact of cinnamic acid on hypertensive left ventricular hypertrophy.

**Results:**

The results showed that cinnamic acid lowered blood pressure and attenuated left ventricular hypertrophic and fibrotic alterations in the ang II-infused mice. Cinnamic acid counteracted hypertrophic responses, impairment of the mitochondrial function and overproduction of mitochondrial reactive oxygen species (ROS) in the cardiomyocytes exposed to ang II. At the molecular level, cinnamic acid mitigated ang II-induced activation of signal transducer and activator of transcription 3 (STAT3) and extracellular signal-regulated kinase 1/2 (ERK1/2) in cardiomyocytes. Additionally, cinnamic acid blunted STAT3 and ERK1/2 activation as well as the hypertrophic responses in cardiomyocytes exposed to interleukin 6 (IL-6) as well.

**Conclusion:**

In summary, this is the first study demonstrating that cinnamic acid is effective at mitigating hypertensive left ventricular hypertrophy. Cinnamic acid antagonizes the vasopressor activity of ang II at the systemic level and the ligand-dependent pro-hypertrophic signaling of ang II in cardiomyocytes. Furthermore, our present study presents new evidence supporting that cinnamic acid lessens the activation of STAT3 and ERK1/2, which may in part contribute to its anti-hypertrophic actions in cardiomyocytes.

## 1 Introduction

Hypertension is one of the most important risk factors of cardiovascular diseases and heart failure. Based on the World Health Organization statistics in 2023, approximately 1.28 billion adults worldwide have been diagnosed with hypertension (https://www.who.int/news-room/fact-sheets/detail/hypertension). The burden of hospitalization and mortality of cardiovascular events and heart failure in hypertensive patients remains unabated even with existing antihypertensive treatments (Roger, 2021). Left ventricular hypertrophy develops in approximately 40% of hypertensive patients and significantly contributes to the pathogenesis of adverse cardiovascular events and heart failure under hypertensive conditions (Messerli and Schmieder, 1986; Cuspidi et al., 2012). Therapies mitigating the hypertrophic pathologies in left ventricles may help to decelerate the progression of heart failure and lower the incidence of cardiovascular events in hypertensive patients (Schiattarella and Hill, 2015; Aronow, 2017). Renin-angiotensin system, the most recognized neurohormonal mechanism in the pathogenesis of hypertension, plays vital roles in the pathophysiological alterations of the cardiovascular system. As the main executor of the renin-angiotensin system, angiotensin II (ang II) exerts intrinsic ligand-dependent pro-hypertrophic effects as well as vasopressor activities-associated mechanical stress-related effects on cardiomyocytes, both of which mechanistically underpin the pathological hypertrophic remodeling in the left ventricle. Therefore, therapies mitigating aberrantly elevated ang II signaling hold promise to protect against hypertensive left ventricular hypertrophy.

Natural products open up new opportunities for the development of new drug therapies (Atanasov et al., 2021). Cinnamic acid, a naturally occurring monocarboxylic acid with low toxicity (Hoskins, 1984), is abundantly present in many herbal medicinals including Cinnamomi ramulus and Cinnamomum cassia, which are used in the clinical practice of traditional Chinese medicine for the treatment of hypertension and heart failure (Chen et al., 2021; Mohammadabadi and Jain, 2024; Qu et al., 2023). It has been reported that cinnamic acid attenuates isoproterenol-induced myocardial injuries (Song et al., 2013), alleviates myocardial ischemia/reperfusion injuries (Luan et al., 2022), mitigates doxorubicin-induced myocardial toxicities (Koczurkiewicz-Adamczyk et al., 2021) and reduces diabetes-associated glucotoxicity in cardiomyocytes (Nair et al., 2022; Anupama et al., 2018). Our previous work has also uncovered that cinnamic acid directly suppresses phenylephrine-stimulated hypertrophic phenotype in cardiomyocytes and offsets the pro-hypertrophic impact of transverse aortic constriction (TAC)-induced pressure overload on the heart (Cui et al., 2023). Cinnamic acid also exerts endothelium-dependent vasodilatory effects *ex vivo*, which suggests possible implications of cinnamic acid in mitigating hypertension (Kang et al., 2013). However, it is yet to be determined if cinnamic acid exerts any effects on ang II-mediated hypertensive left ventricular hypertrophy. Moreover, it remains to be elucidated if the pro-hypertrophic ang II signaling in cardiomyocytes could be therapeutically modulated by cinnamic acid. Thus, the current study explored the pharmacological implications of cinnamic acid in ang II-mediated hypertensive left ventricular hypertrophy.

## 2 Materials and methods

### 2.1 Animals and treatments

C57BL/6J mice (male, 7 weeks old, 20 ± 1 g) were ordered from Shanghai Laboratory Animal Research Center (Shanghai, China). The mice were maintained in standard laboratory conditions and had free access to water and food. The animal handling was performed in compliance with the National Institutes of Health guide and approved by the Institutional Animal Care and Use Committee of Yueyang Hospital of Integrated Traditional Chinese Medicine, Shanghai University of Traditional Chinese Medicine (Approval No. YYLAC-2019-018). Ang II (Sigma-Aldrich, United States) was prepared in sterile saline solution. To induce hypertension and left ventricular hypertrophy, micro-osmotic pumps (Alzet MODEL 1002, United States) loaded with ang II were subcutaneously implanted with the infusion parameter set at 1 μg/kg/min for 14 days, a dose and duration regimen selected with reference to the published protocols (Kurisu et al., 2003; Wang et al., 2021; Wang et al., 2018). Ang II-infused mice received daily gavage of either vehicle (i.e., 0.5% sodium carboxymethyl cellulose solution) or cinnamic acid (Shanghai Shifeng Biological Technology CO., Ltd., China) at 60 and 300 mg/kg for 14 days. The doses were selected primarily based on our previous findings on the effects of cinnamic acid against TAC-induced left ventricular hypertrophy (Cui et al., 2023). The pumps loaded with saline solution were also implanted to the vehicle-treated mice (sham controls). The indicated treatments started at 24 h after the initiation of ang II infusion. The animals were subjected to blood pressure measurement before ang II infusion, 1 week after ang II infusion and at the end of the 14-day ang II infusion. At the end of the indicated treatments, the mice were euthanized. The heart weight and the length of the tibia were then measured to assess the heart index. Afterward, the left ventricles were prepared for paraffin sectioning to obtain cross-sections for histological and immunohistochemical examinations.

### 2.2 Assessment of the blood pressure

BP-2010A instrument (Softron Biotechnology, China) was used to measure the blood pressure following the protocol described in our previous work (Wu et al., 2019; Ding et al., 2019). In brief, the mouse was restrained under conscious state in a 35°C warming chamber and allowed to stabilize for 5–10 min, followed by securing the cuff around the tails. Inflation and deflation of the tail cuff were repeated multiple times to precondition the animal, followed by recording systolic blood pressure (SBP) and diastolic blood pressure (DBP). Multiple readings were acquired and minimally 5 readings within the 5–10 mmHg range were averaged to obtain the SBP or DBP for each mouse.

### 2.3 Myocardial fibrosis and cardiomyocyte hypertrophy evaluation

After fixation in 4% paraformaldehyde for 24 h, left ventricles were subjected to further processing to prepare paraffin sections (4-µm thick) for Masson’s trichrome staining and immunohistochemical assessment. Sections were also sequentially probed with anti-natriuretic peptides A (ANP) antibody (1:500, ab225844, Abcam, United States) at 4°C overnight and Cy3-conjugated anti-rabbit secondary antibody (1:500, C2306, Sigma-Aldrich, United States) at room temperature for 1 h in the dark. Wheat-germ agglutinin (WGA) expression pattern was examined using Alexa5 Fluor 488-conjugated WGA antibody (10 μg/mL, W11261, Thermo Fisher Scientific, United States) at room temperature for 30 min in the dark. The images were recorded using a light microscope (DM 2000, Leica, Germany) or a fluorescence microscope (DM6000B, Leica, Germany). Quantification analysis was performed using ImageJ.

### 2.4 Cardiomyocyte culture and treatments

Isolation of the primary neonatal rat cardiomyocytes (NRCMs) was performed using the heart dissected from 2- to 3-day old Sprague-Dawley rats (Shanghai Laboratory Animal Center, Shanghai, China) following previously described methods (Cui et al., 2023). In brief, the hearts were washed in HBSS and minced into pieces in the size of 1–2 mm^3^. Next, the minced tissue was digested with 0.25% Trypsin-EDTA (Gibco, United States) at 4°C overnight. The following day, 1.5 mg/mL collagenase II (Thermo Fisher Scientific, United States) digestion was performed by incubating and shaking the tissue at 37°C for 5 min. This serial enzyme digestion procedure was repeated 5 to 7 times. Cell suspension was collected and filtered through a 100-mesh cellular sifter, followed by centrifugation at 800 rpm for 5 min to harvest the cells. The cells were then resuspended and plated in DMEM containing 5% FBS and 1% penicillin/streptomycin (Thermo Fisher Scientific, United States) for 2 h, followed by re-seeding of the nonattached cardiomyocytes into 24-well plates at 6 × 10^4^ cells/well for immunocytochemistry experiments or 12-well plates at 4 × 10^5^ cells/well for Western blotting and real-time quantitative polymerase chain reaction (qPCR) analyses.

H9c2 cells (Chinese Academy of Sciences Cell Bank of Type Culture Collection, Shanghai, China) were maintained in DMEM containing 10% FBS and 1% penicillin/streptomycin. For Western blotting, H9c2 cells seeded in 6-well plates at 6.4 × 10^5^ cells/well were stimulated with recombinant IL-6 (IL-6, Sino Biological Lnc, China). For immunocytochemistry and mitochondrial analyses, H9c2 cells were plated in 24-well plates at 2 × 10^4^ cells/well and 1 × 10^4^/well, respectively.

### 2.5 Immunocytochemistry

After fixation in 4% paraformaldehyde for 15 min, permeabilization in 0.1% Triton X-100 for 10 min and blocking in 1% BSA for 30 min at room temperature, rhodamine phalloidin (100 nM, CA1610, Solarbio, China) or rabbit polyclonal anti-ANP antibody (1:750, ab14348, Abcam, United States) staining was performed at 4°C overnight. Cy3-conjugated anti-rabbit secondary antibody (1:500, C2306, Sigma-Aldrich, United States) was subsequently incubated at room temperature for 1 h in the dark. The nuclei were visualized by 4′,6-diamidino-2-phenylindole (DAPI). A fluorescence microscope (DMI6000, Leica, Germany) was employed for the imaging purpose. ImageJ was used for quantification analysis.

### 2.6 Real-time qPCR

Total RNA from NRCMs was extracted using miRNeasy Mini Kit (Qiagen, Germany). Reverse transcription and real-time PCR reactions were set up using PrimeScript RT Master Mix (TaKaRa, Japan) and LightCycler 480 SYBR Green I Master (Roche, Germany), respectively. The PCR was run on a LightCycler 480 II (Roche, United States). The primer sequences (5′-3′) are as follows. Rat natriuretic peptide type B (Nppb), forward primer: GAA​CAA​TCC​ACG​ATG​CAG​AAG​C, reverse primer: TCT​GCC​CAA​AGC​AGC​TTG​AA; rat Gapdh, forward primer: ACA​GCA​ACA​GGG​TGG​TGG​AC, reverse primer: TTT​GAG​GGT​GCA​GCG​AAC​TT. The fold change in the expression of Nppb was calculated based on 2−[Ct (Nppb)-Ct (Gapdh)].

### 2.7 Western blotting

RIPA buffer (Beyotime, China) containing protease inhibitors and phosphatase inhibitors (Roche, Germany) was used to obtain cell lysate. The protein samples were run in 10% SDS-PAGE gels and transferred to polyvinylidene fluoride membranes (Millipore, United States). The membranes were blocked with 5% BSA for 1 h at room temperature and incubated overnight at 4°C with primary antibodies including monoclonal mouse anti-STAT3 antibody (1:1000, 9139, Cell Signaling Technology, United States), monoclonal mouse anti-phosphorylated-STAT3 (pSTAT3) antibody (1:2000, 4,113, Cell Signaling Technology, United States), polyclonal rabbit anti-ERK1/2 antibody (1:1000, 9102, Cell Signaling Technology, United States), monoclonal mouse anti-phosphorylated ERK1/2 (pERK1/2) antibody (1:2000, 9106, Cell Signaling Technology, United States) or monoclonal mouse anti-alpha tubulin (α-tubulin) antibody (1:4500, ab7291, Abcam, United States). After primary antibody incubation, the secondary antibodies were applied for 1 h at room temperature, which included horseradish peroxidase-conjugated goat anti-mouse (1:3000, W4028, Promega, United States) and anti-rabbit secondary antibody (1:3000, W4011, Promega, United States). The signal was detected using WesternBright ECL reagent (Advansta, United States) and recorded by an UVP imaging system (BioSpectrum 410, United States). ImageJ was used for densitometry analysis.

### 2.8 Mitochondrial analyses

Mitochondrial Membrane Potential Assay Kit (C2006, Beyotime, China) was adopted to analyze the mitochondrial membrane potential (MMP) in H9c2 cells following the manufacturer’s instructions. Briefly, the cells were incubated with the JC-1 detection solution at 37°C for 20 min in the dark. After incubation with the JC-1 detection reagent, the fluorescent signals indicative of JC-1 aggregates (in red) and JC-1 monomers (in green) were recorded. Mitochondrial Permeability Transition Pore Assay kit (C2009S, Beyotime, China) was employed to assess the opening of mitochondrial permeability transition pore (mPTP) in H9c2 cells following the manufacturer’s protocol. In brief, the cells were incubated in calcein acetoxymethyl ester staining solution and CoCl2 fluorescence quenching solution at 37°C for 50 min in the dark. MitoSOX (M36008, Thermo Fisher Scientific, United States) was applied to detect mitochondrial production of reactive oxygen species (ROS) in H9c2 cells. In brief, the cells were stained with 5 µM MitoSOX at 37°C for 10 min in the dark. The nuclei were counterstained with DAPI. Images were captured by a fluorescence microscope (DMI6000, Leica, Germany). The fluorescence was analyzed by ImageJ.

### 2.9 Statistical analysis

The data were presented as mean ± standard error of mean (SEM). One-way ANOVA with the Tukey multiple-comparisons test was conducted for the statistical analyses. Conclusions of statistically significance were reached when P < 0.05.

## 3 Results

### 3.1 Cinnamic acid lowers blood pressure and alleviates left ventricular hypertrophy in the ang II-infused mice

**FIGURE 1:**
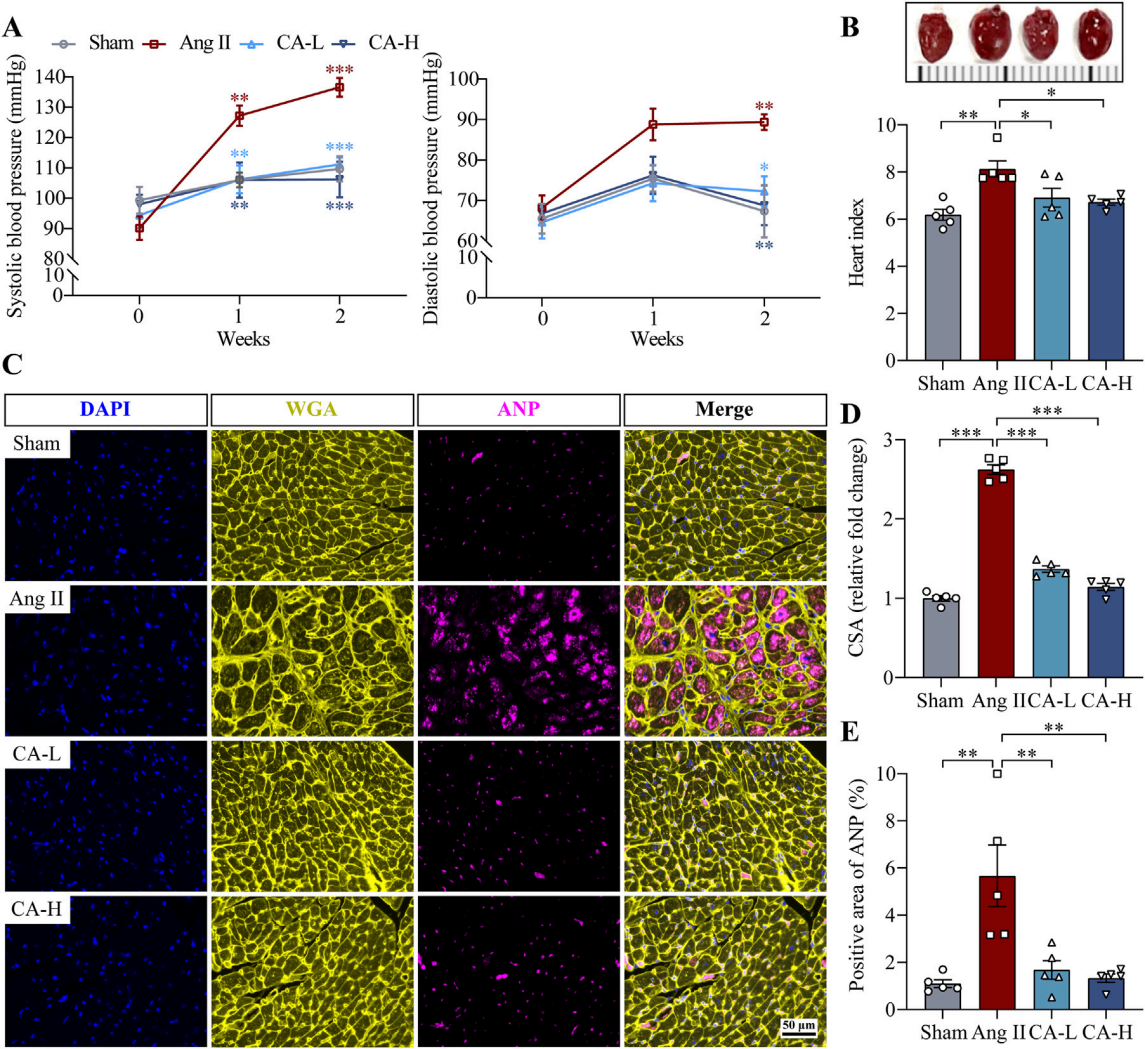
Cinnamic acid mitigates hypertension and hypertrophic pathologies in the left ventricles in ang II-infused mice. The mice infused with ang II were treated with vehicle (Ang II) or cinnamic acid at 60 mg/kg (CA-L) and 300 mg/kg (CA-H) for 14 days. Sham controls received vehicle treatment (n = 5 per group). **(A)** SBP and DBP readings. **(B)** Photographed hearts (upper panel) and the heart index (lower panel). **(C)** The pattern of WGA (in yellow) and ANP (in pink) in the left ventricles. DAPI (in blue) highlighted the nuclei. Scale bar, 50 μm. **(D)** CSA of cardiomyocytes. **(E)** ANP immunopositivity. *P < 0.05, **P < 0.01, ***P < 0.001. Ang II, angiotensin II; CA, cinnamic acid; CSA, cross-sectional area.

**FIGURE 2:**
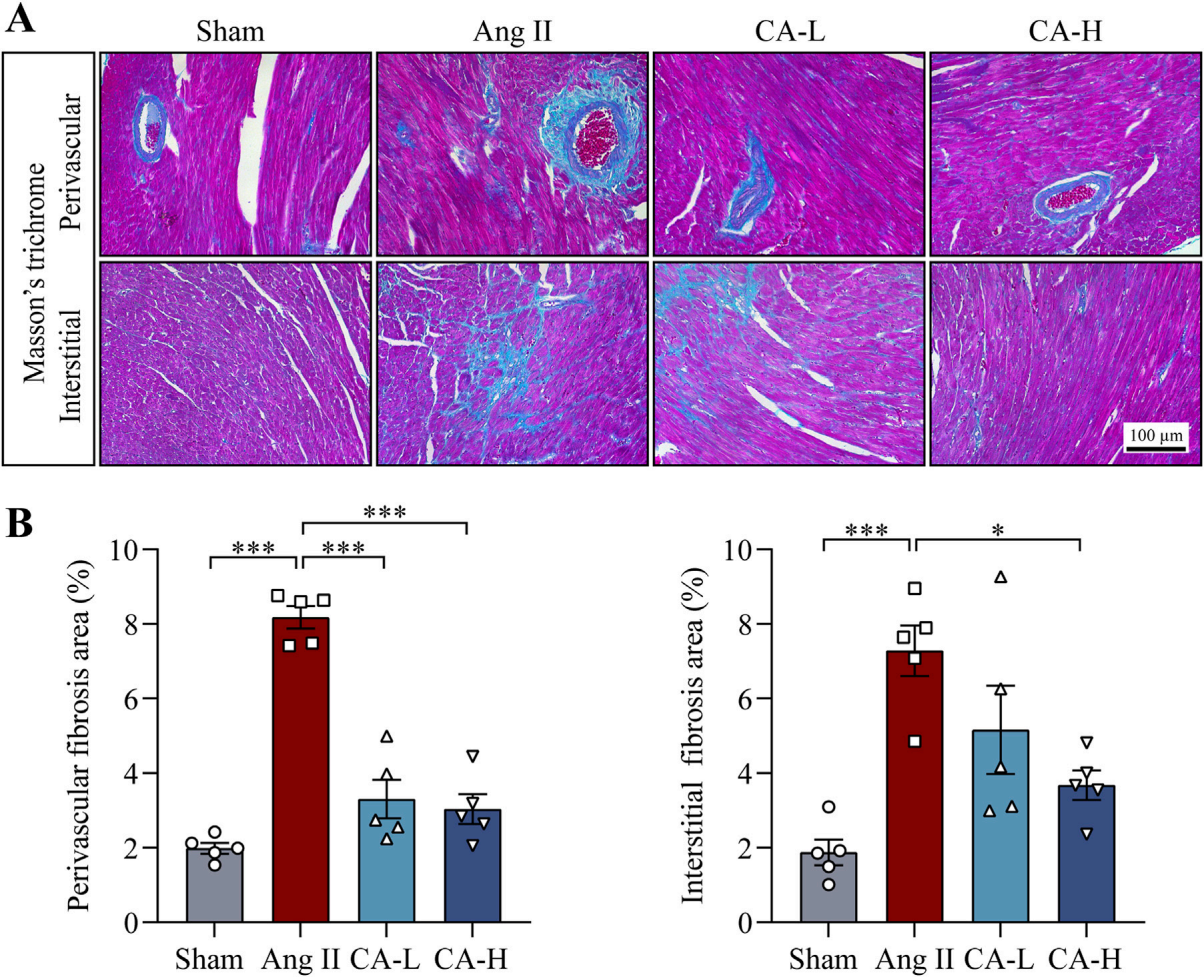
Cinnamic acid alleviates fibrotic changes in the left ventricles from the ang II-infused mice. The mice infused with ang II received vehicle (Ang II) or cinnamic acid treatment at 60 mg/kg (CA-L) and 300 mg/kg (CA-H) for 14 days. Sham controls received vehicle treatment in the same manner (n = 5 per group). **(A)** Masson’s trichrome-stained images. Scale bar, 100 μm. **(B)** Perivascular and interstitial fibrotic areas (%). *P < 0.05, ***P < 0.001. Ang II, angiotensin II; CA, cinnamic acid.

The impact of cinnamic acid on hypertensive left ventricular hypertrophy was directly examined in ang II-infused mice *in vivo*. Significantly elevated SBP and DBP were observed in the mice infused with ang II compared to the sham controls. In contrast, SBP and DBP were decreased in the mice infused with ang II and treated with 60 and 300 mg/kg cinnamic acid (Figure 1A). Compared to the sham controls, the heart index (i.e., the ratio of the heart weight to the tibia length) was significantly higher in the ang II-infused mice, whereas lower heart index was found in the cinnamic acid-treated ang II-infused mice (Figure 1B). Moreover, the cardiomyocytes in the left ventricles were significantly enlarged in the ang II-infused mice. However, the cardiomyocyte was remarkably smaller in the cinnamic acid-treated ang II-infused mice (Figures 1C, D). Meanwhile, the level of ANP in the left ventricular cardiomyocytes was notably increased as a result of ang II infusion. In contrast, the level of the cardiomyocyte ANP was markedly decreased in the cinnamic acid-treated ang II-infused mice (Figures 1C, E). In addition to the hypertrophic responses in cardiomyocytes, ang II infusion also leads to myocardial fibrosis, another hallmark feature of pathological cardiac remodeling responsible for irreversible progression of cardiac dysfunction (Schnee and Hsueh, 2000). Consistently, Masson’s trichrome staining revealed prominent left ventricular perivascular and interstitial fibrosis from the ang II-infused mice. In contrast, the fibrotic pathologies in the perivascular region were attenuated in ang II-infused mice treated with both lower dose (i.e., 60 mg/kg) and higher dose (i.e., 300 mg/kg) of cinnamic acid. Meanwhile, interstitial fibrosis in the left ventricles was markedly alleviated in the ang II-infused mice that received treatment of 300 mg/kg cinnamic acid. A modest yet statistically insignificant reduction of interstitial fibrosis was also noted as a result of 60 mg/kg cinnamic acid treatment (Figure 2). The blood pressure and morphological assessments indicate that cinnamic acid attenuates ang II-mediated hypertension as well as hypertrophic and fibrotic pathologies in the left ventricle.

### 3.2 Cinnamic acid directly counteracts ang II-induced hypertrophic responses in cardiomyocytes

**FIGURE 3:**
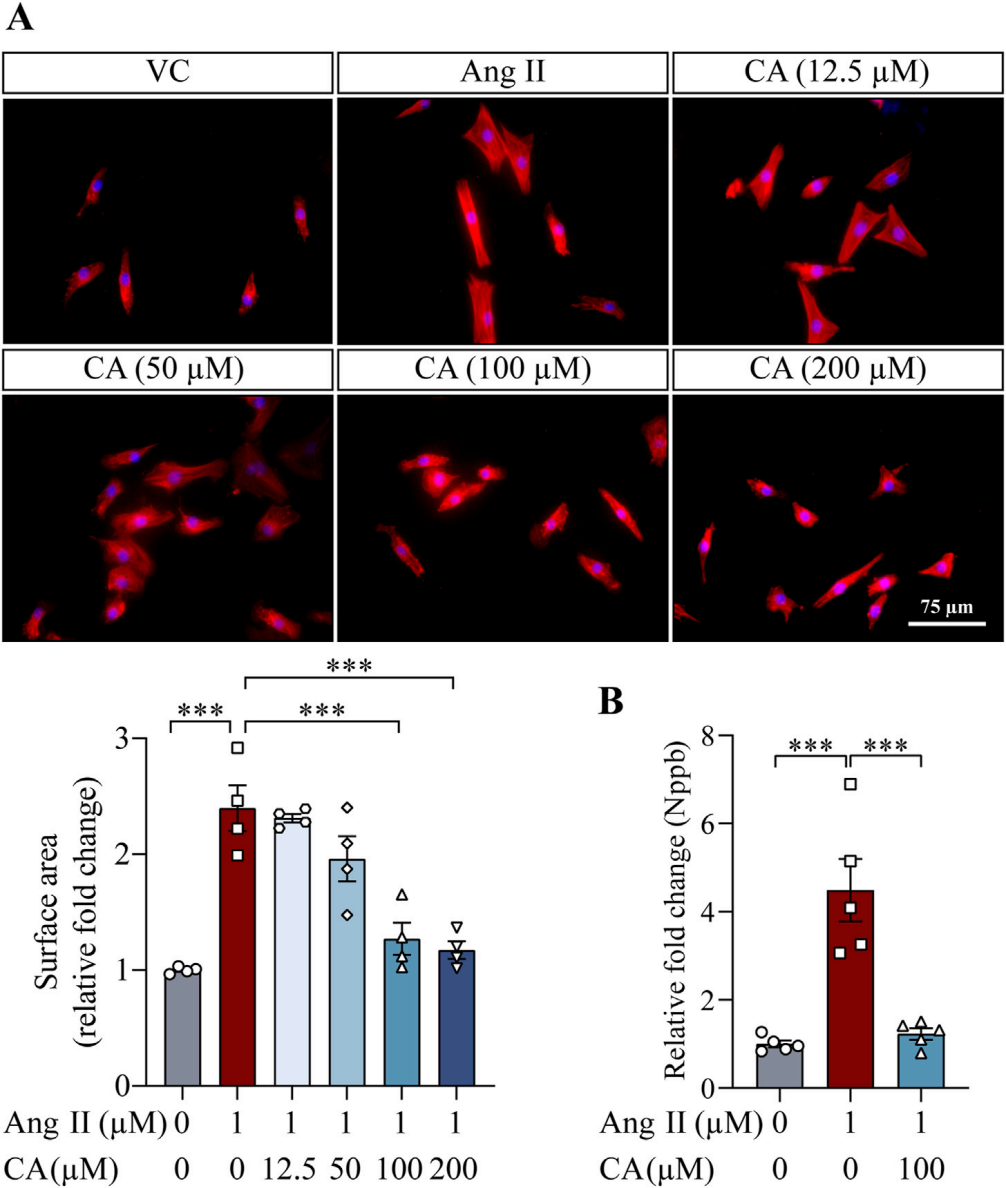
Cinnamic acid mitigates hypertrophic responses in the primary cardiomyocytes exposed to ang II. **(A)** After 30-min incubation with vehicle or cinnamic acid at the specified doses, NRCMs were exposed to ang II for 24 h and subjected to Rhodamine phalloidin (in red) staining (n = 4 per group). Over 100 cells per group were examined to estimate the size of NRCMs. DAPI (in blue) highlighted nuclei. Scale bar, 75 μm. **(B)** After 30-min incubation with vehicle or cinnamic acid at 100 μM, NRCMs were exposed to ang II at 1 µM for 24 h. The gene expression of Nppb was then analyzed (n = 5 per group). ***P < 0.001. Ang II, angiotensin II; CA, cinnamic acid; VC, vehicle control.

**FIGURE 4:**
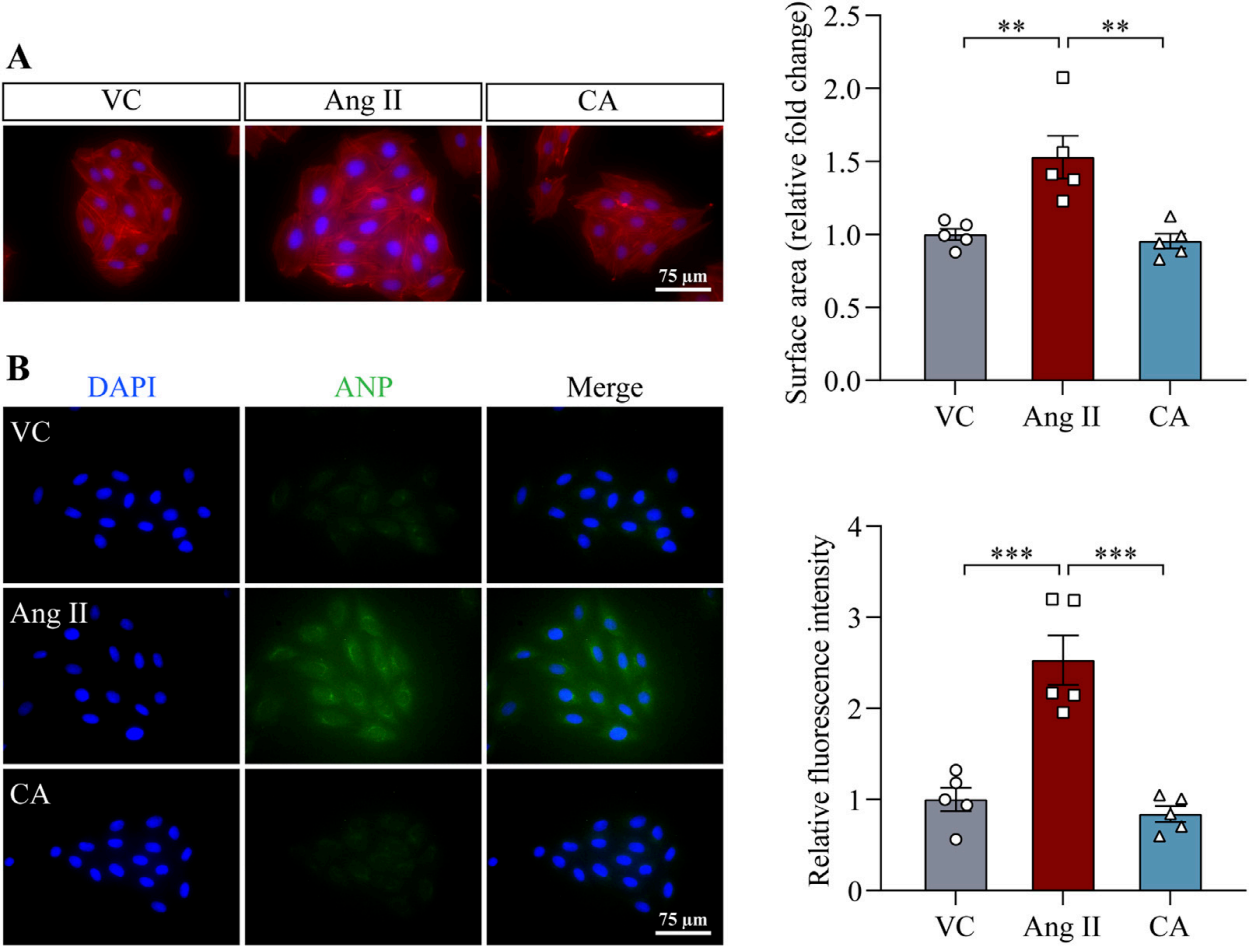
Cinnamic acid attenuates hypertrophic responses in ang II-exposed H9c2 cells. After 30-min incubation with vehicle or cinnamic acid at 100 μM, H9c2 cells were exposed to ang II at 1 µM for 48 h (n = 5 per group). **(A)** Rhodamine phalloidin (in red) and DAPI (in blue) staining. Scale bar, 75 μm. The size of H9c2 cells was quantified from ≥125 cells per group. **(B)** Immunostaining of ANP (in red) and DAPI staining (in blue). Scale bar, 75 μm **P < 0.01, ***P < 0.001. Ang II, angiotensin II; CA, cinnamic acid; VC, vehicle control.

The findings above demonstrate that along with lowering the blood pressure, cinnamic acid attenuates left ventricular hypertrophy in the ang II-infused mice. Elevated blood pressure causes pressure overload and the ensuing mechanical stress on cardiomyocytes in part contributes to the pathogenesis of hypertrophic remodeling of the left ventricles in hypertensive patients (Blaustein, 2017). On the other hand, in addition to vasoconstrictor-associated mechanical stress dependent mechanisms, ang II directly stimulates hypertrophic responses in cardiomyocytes, resulting in hypertrophic pathologies in the heart (Zablocki and Sadoshima, 2013). Thus, it remains to be determined whether the effect of cinnamic acid against the development of ang II-induced hypertrophic phenotypes *in vivo* results from decreased blood pressure or implicates direct actions that offset the ligand-dependent pro-hypertrophic activities of ang II in cardiomyocytes. In order to further delineate the potential impact of cinnamic acid on the ligand-dependent action of ang II on cardiomyocytes, primary NRCMs were subjected to ang II stimulation, followed by assessments of the effects of cinnamic acid on the hypertrophic responses in the cells. Rhodamine phalloidin staining of the filamentous actin showed that the size of NRCMs was increased in response to ang II exposure. However, dose-dependent effects of cinnamic acid against ang II-induced hypertrophic responses in NRCMs were observed. The cardiomyocytes were significantly smaller as a result of 100 and 200 μM cinnamic acid treatment, which was not seen in the cells treated with 12.5 or 50 μM cinnamic acid (Figure 3A). Real-time qPCR analyses further showed that Nppb gene expression was significantly upregulated when the cells were exposed to ang II. In contrast, much lower expression of Nppb was detected in the ang II-exposed NRCMs treated with 100 μM cinnamic acid (Figure 3B). Similar observations were also made in H9c2 cardiomyocytes. Cinnamic acid delivered at 100 μM counteracted ang II-induced increases in the size (Figure 4A) and intracellular level of ANP in H9c2 cells (Figure 4B). The results above collectively support that cinnamic acid directly mitigates the ligand-dependent effect of ang II on cardiomyocyte hypertrophy.

### 3.3 Cinnamic acid alleviates ang II-triggered mitochondrial dysfunction and ROS production in cardiomyocytes

**FIGURE 5:**
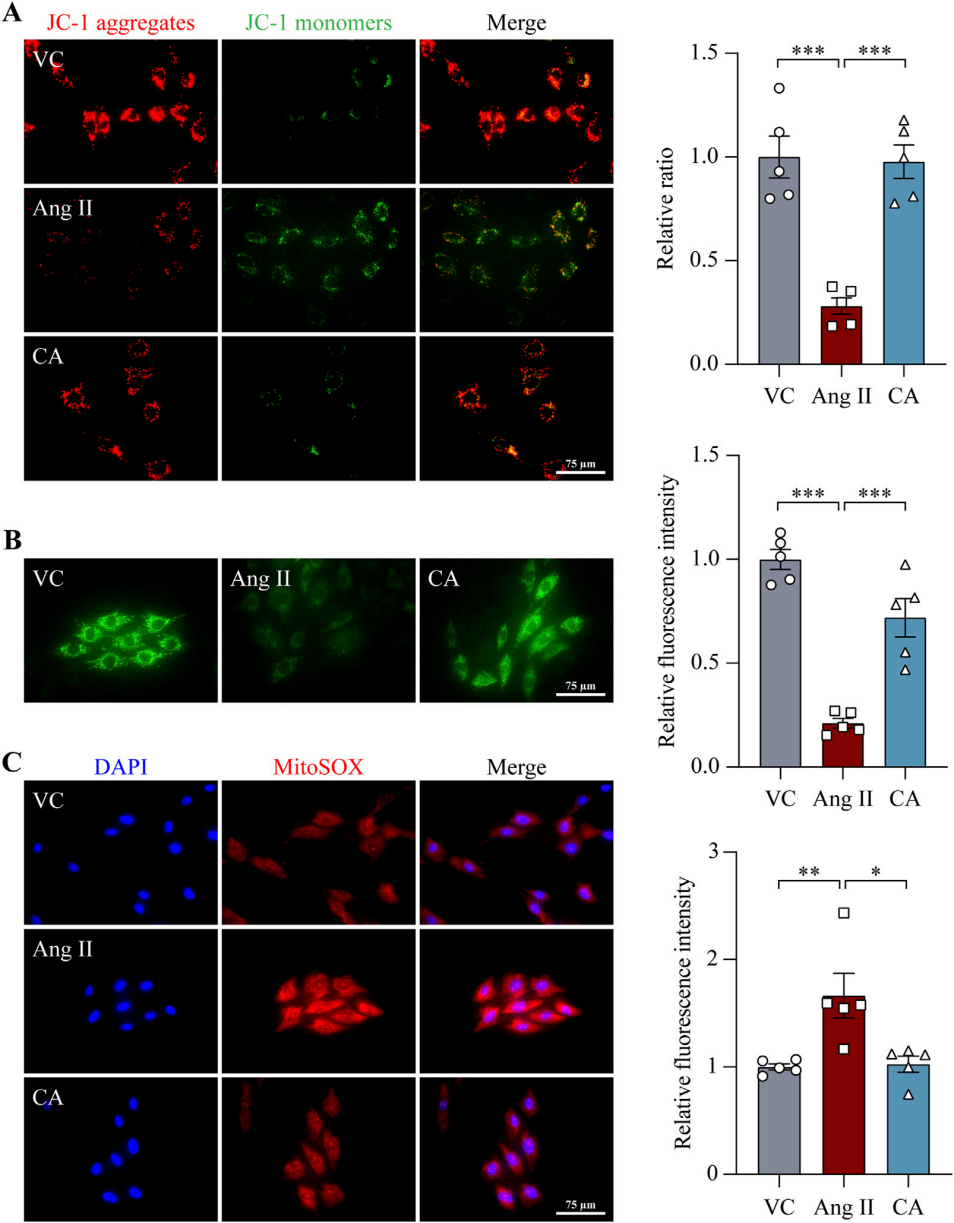
Cinnamic acid maintains mitochondrial function and diminishes mitochondrial superoxide overproduction in ang II-exposed cardiomyocytes. After 30-min incubation with vehicle or cinnamic acid (100 μM), H9c2 cells were subjected to 48-h exposure to ang II (n = 5 per group). **(A)** MMP evaluation (red, J-aggregates; green, J-monomers). Scale bar, 75 μm. **(B)** mPTP opening assessment by calcein (in green). Scale bar, 75 μm. **(C)** Mitochondrial superoxide assessment by MitoSOX Red (in red). DAPI (in blue) highlighted the nuclei. Scale bar, 75 μm *P < 0.05, **P < 0.01, ***P < 0.001. Ang II, angiotensin II; CA, cinnamic acid; VC, vehicle control.

Mitochondrial dysfunction and associated ROS overproduction not only are hallmark features of hypertrophic and failing cardiomyocytes, but also actively promotes the pathological changes associated with hypertrophic remodeling in the heart and transition to heart failure (Osterholt et al., 2013). Ang II directly stimulates the production of mitochondrial ROS, which is mechanistically implicated in the hypertrophic responses in cardiomyocytes (Dai et al., 2011). Therefore, mitochondrial function was further examined. MMP was noticeably impaired in the H9c2 cardiomyocytes upon ang II exposure. On the contrary, improved MMP was noted in the ang II-exposed cells treated with 100 μM cinnamic acid (Figure 5A). Meanwhile, ang II caused marked opening of mPTP in H9c2 cells, whereas 100 μM cinnamic acid treatment counteracted the deleterious impact of ang II on mPTP (Figure 5B). Furthermore, MitoSOX Red labeling of superoxide in mitochondria uncovered that the superoxide level was elevated in the cells exposed to ang II. However, the level of mitochondrial superoxide was remarkably decreased in the cells treated with 100 μM cinnamic acid (Figure 5C). The mitochondrial functional assays thus reveal that the anti-hypertrophic action of cinnamic acid implicates attenuating ang II-triggered mitochondrial impairment in cardiomyocytes.

### 3.4 Cinnamic acid mitigates ang II-induced activation of STAT3 and ERK1/2 in cardiomyocytes

**FIGURE 6:**
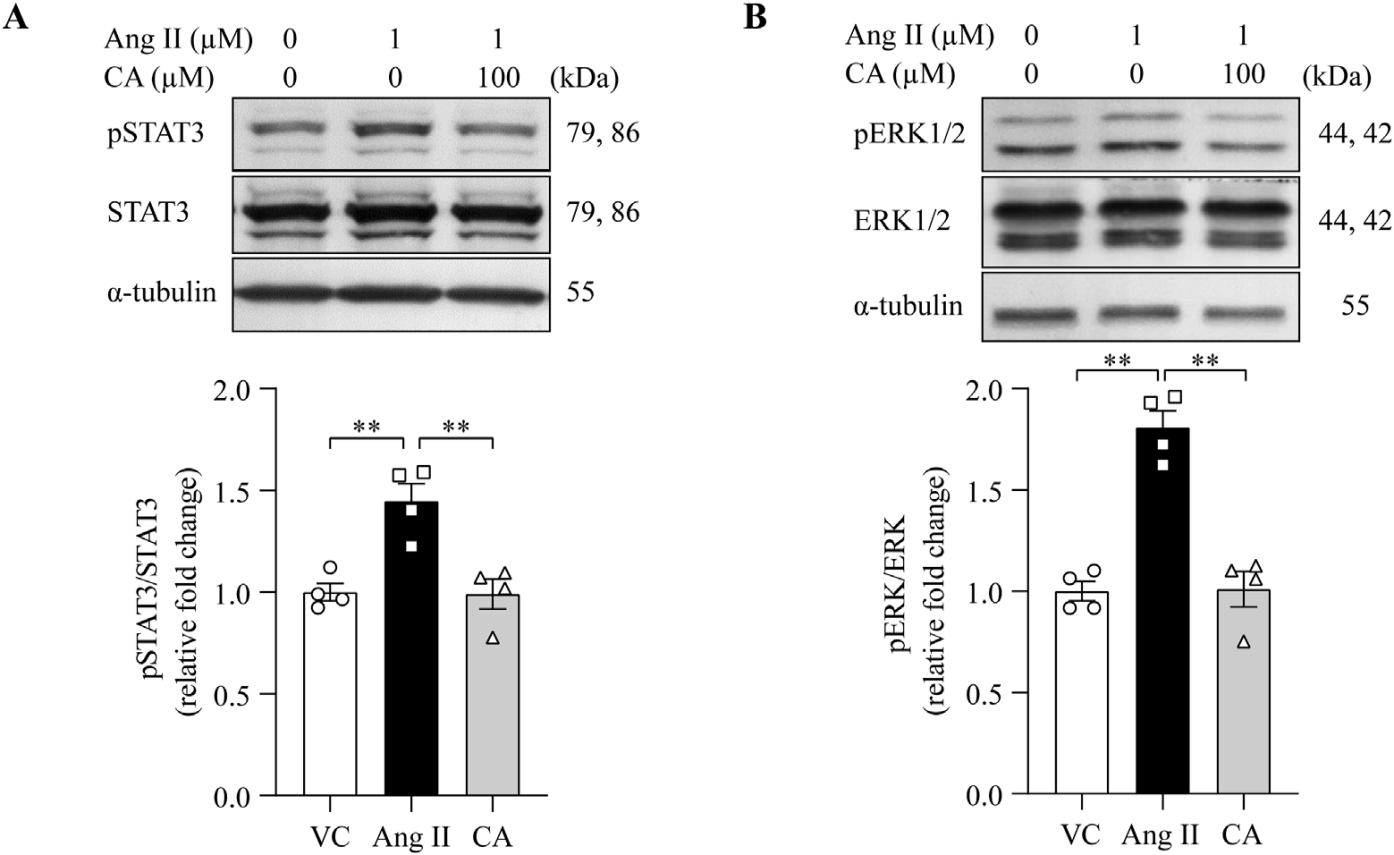
Cinnamic acid dampens cardiomyocyte STAT3 and ERK1/2 activation in response to ang II. After 30-min incubation with vehicle or cinnamic acid, NRCMs were exposed to ang II for 12 h **(A)** or 5 min **(B)**. **(A)** The levels of pSTAT3 and STAT3. **(B)** The levels of pERK1/2 and ERK1/2. α-tubulin was included for normalization purposes. n = 4 per group. **P < 0.01. Ang II, angiotensin II; CA, cinnamic acid; VC, vehicle control.

The pro-hypertrophic responses of ang II in cardiomyocytes are mediated by multiple downstream signaling mechanisms. For example, enhanced activation of STAT3 relays the hypertrophic responses of ang II and participates in ang II-triggered mitochondrial impairment (Han et al., 2018). ERK1/2 activation contributes to cardiomyocyte hypertrophy in the setting of ang II exposure (Bueno and Molkentin, 2002). Therefore, the level of phosphorylated STAT3 (pSTAT3) and ERK1/2 (pERK1/2) was assessed to gain molecular understanding of the impact of cinnamic acid on ang II-stimulated pro-hypertrophic signaling in cardiomyocytes. The level of pSTAT3 in cardiomyocytes was increased in response to ang II exposure, whereas markedly lower level of pSTAT3 was seen in the cells treated with 100 μM cinnamic acid (Figure 6A). Upon ang II exposure, phosphorylation of ERK1/2 was also noted to be elevated. However, 100 μM cinnamic acid treatment resulted in significantly decreased level of pERK1/2 in the ang II-exposed cells (Figure 6B). The analyses here indicate that cinnamic acid mitigates ang II-stimulated activation of pro-hypertrophic signaling in cardiomyocytes.

### 3.5 Cinnamic acid mitigates IL-6-induced activation of STAT3 and ERK1/2 in cardiomyocytes

**FIGURE 7:**
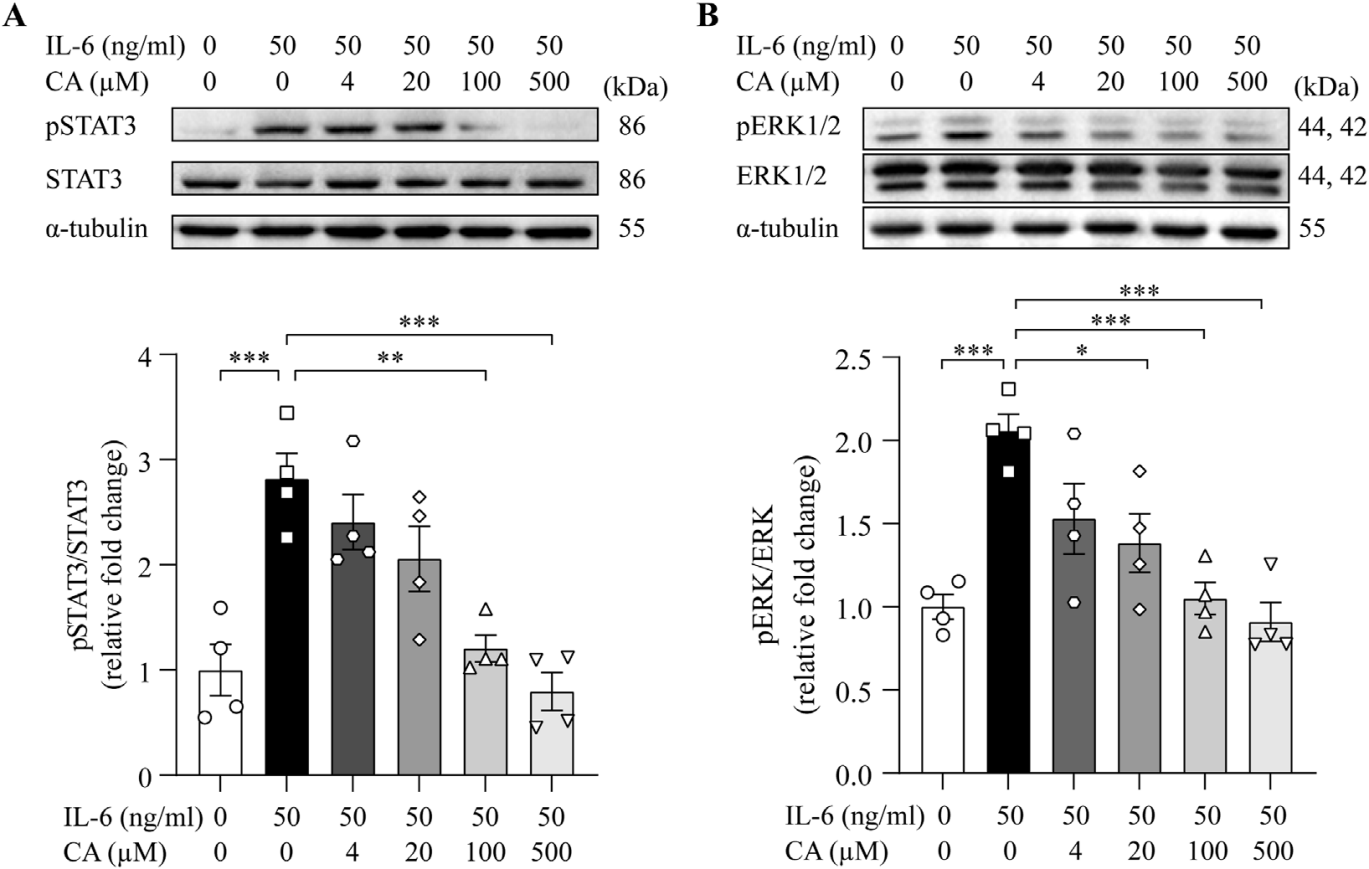
Cinnamic acid dampens cardiomyocyte STAT3 and ERK1/2 activation in response to IL-6 stimulation. After 30-min incubation with vehicle or cinnamic acid, H9c2 cells were exposed to IL-6 for 15 min (n = 4 per group). The levels of pSTAT3 and STAT3 **(A)** as well as pERK1/2 and ERK1/2 **(B)** were then analyzed. α-tubulin was included for normalization purposes. *P < 0.05, **P < 0.01, ***P < 0.001. CA, cinnamic acid.

**FIGURE 8:**
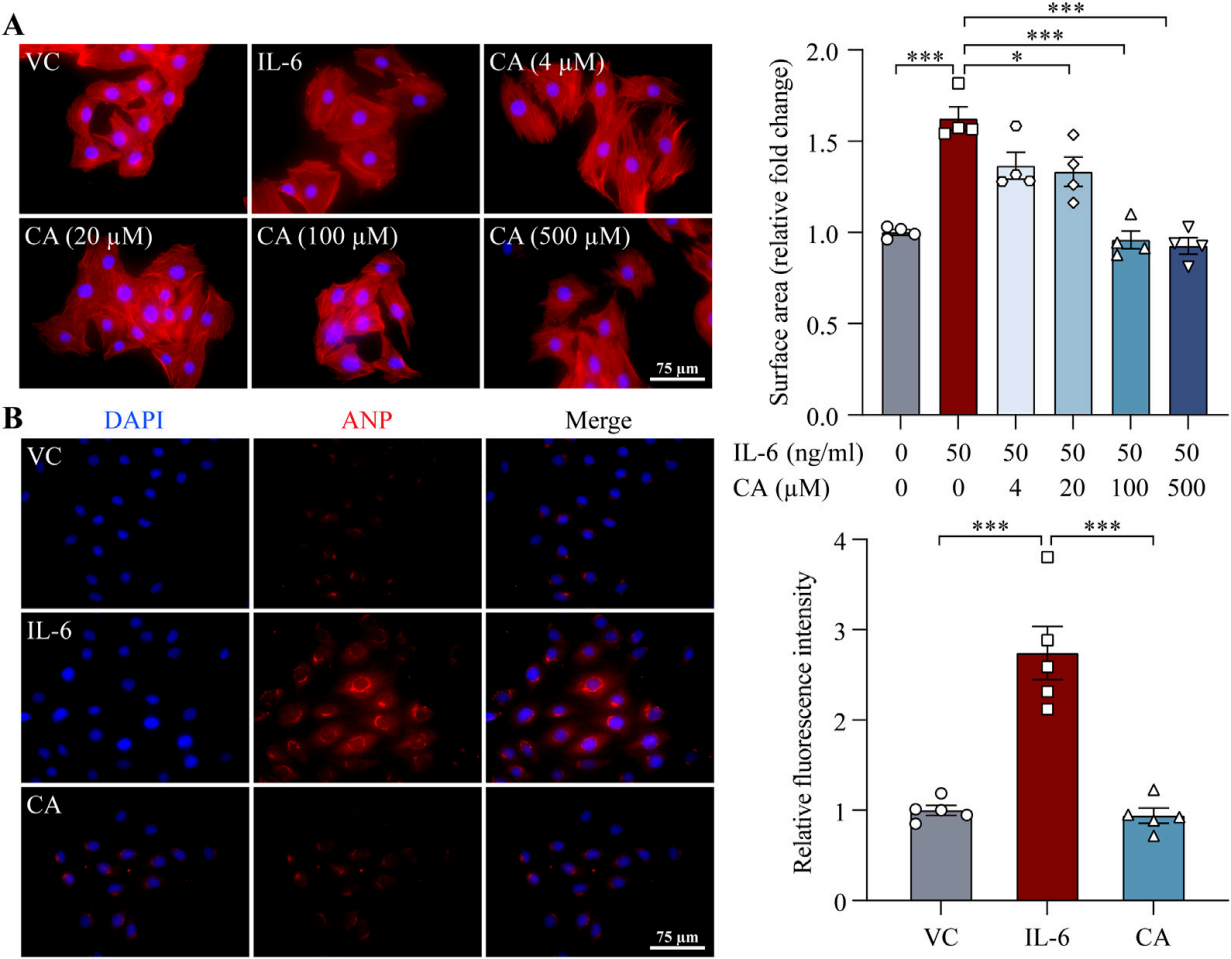
Cinnamic acid mitigates IL-6-induced cardiomyocyte hypertrophy. **(A)** After 30-min incubation with vehicle or cinnamic acid, H9c2 cells were subjected to 24-h stimulation with IL-6 (n = 4 per group). Rhodamine phalloidin (in red) and DAPI (in blue) were subsequently stained. The surface area of H9c2 cells was quantified from over 100 cells per group. Scale bar, 75 μm. **(B)** After 30-min incubation with vehicle or cinnamic acid (100 μM), H9c2 cells were subjected to 24-h incubation with IL-6 (50 ng/mL) (n = 5 per group). ANP (in red) and DAPI (in blue) were stained. Scale bar, 75 μm *P < 0.05, ***P < 0.001. CA, cinnamic acid; VC, vehicle control.

STAT3 and ERK1/2 are important mediators of pro-hypertrophic signaling pathways shared by various pro-hypertrophic stimuli. To better understand the mode of action of cinnamic acid on STAT3-and ERK1/2-mediated pro-hypertrophic signaling in cardiomyocytes, IL-6, which canonically activates STAT3 and ERK1/2 and triggers pro-hypertrophic responses in cardiomyocytes (Meléndez et al., 2010; Hirota et al., 1995), was further applied to the cardiomyocytes. As expected, distinct STAT3 activation was readily noted upon IL-6 exposure. However, cinnamic acid dose-dependently attenuated IL-6-induced STAT3 activation in cardiomyocytes. Significant reductions in the level of pSTAT3 were observed in the cells treated with 100 and 500 μM cinnamic acid but not when cinnamic acid was delivered at 4 or 20 μM (Figure 7A). Similarly, IL-6-stimulated elevation in the level of pERK1/2 was dose-dependently blunted by cinnamic acid as well. Significantly lower levels of pERK1/2 were observed in the cells treated with 20, 100 and 500 μM cinnamic acid but not in those treated with 4 μM cinnamic acid (Figure 7B). Furthermore, the size of H9c2 cells was significantly increased due to IL-6 exposure. However, cinnamic acid treatment dose-dependently reduced the size of the IL-6-exposed H9c2 cells. Significantly smaller cardiomyocytes were noted as a result of treatment with 20, 100 and 500 μM cinnamic acid but not with 4 μM cinnamic acid treatment. Moreover, the anti-hypertrophic effect of cinnamic acid in IL-6-exposed H9c2 cells appeared to reach a plateau at 100 μM (Figure 8A). Consistently, the intracellular level of ANP were significantly increased as a result of IL-6 stimulation, whereas markedly decreased level of intracellular ANP was found in the IL-6-exposed H9c2 cells treated with 100 μM cinnamic acid (Figure 8B). Therefore, along with the above-mentioned impact of cinnamic acid against ang II-triggered STAT3 and ERK1/2 activation, these results corroborate the notion that the mechanisms underpinning the therapeutic action of cinnamic acid in the setting of cardiomyocyte hypertrophy involves mitigating the activation of common mediators of the pro-hypertrophic signaling pathways.

## 4 Discussion

**FIGURE 9:**
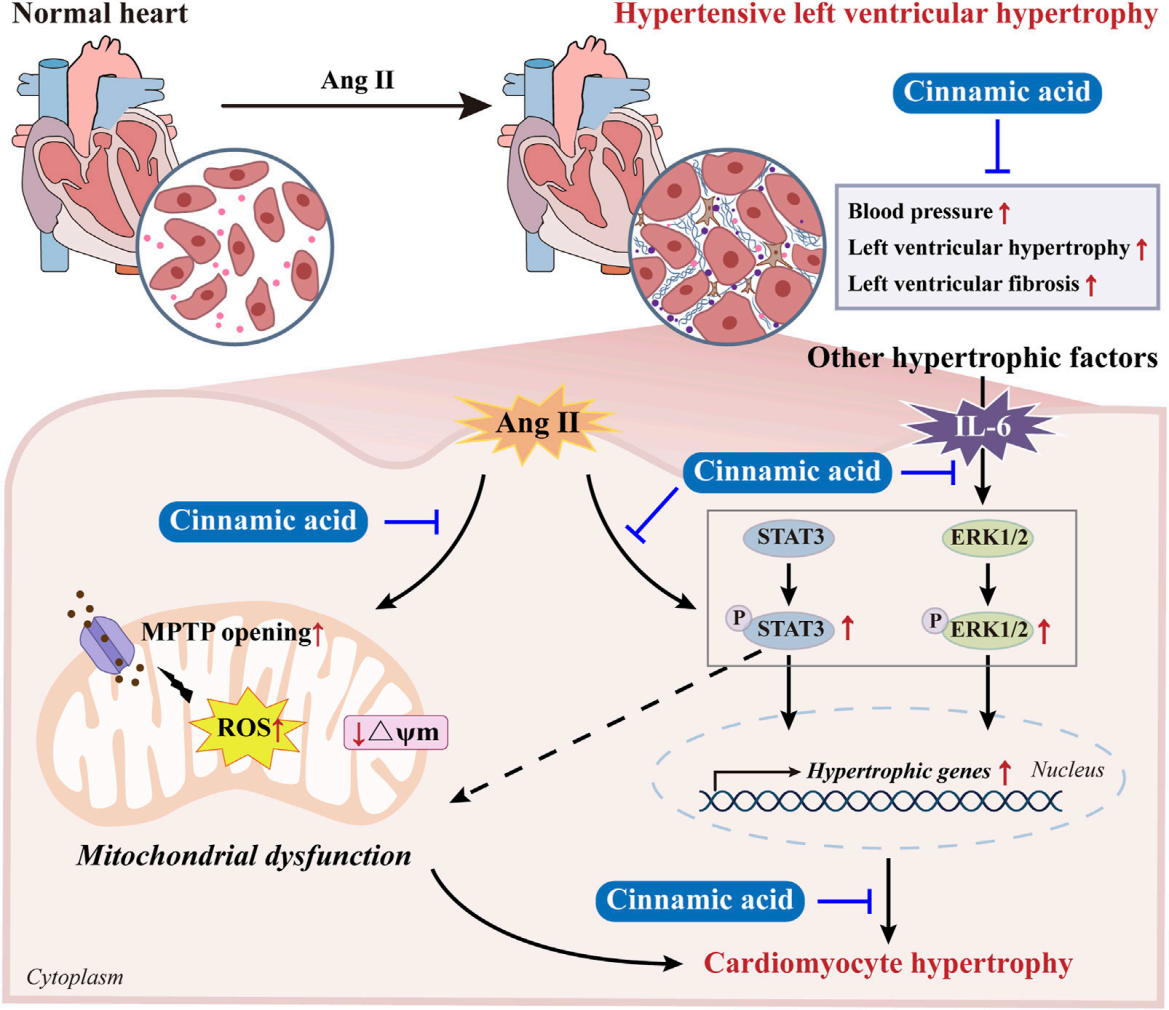
A schematic drawing summarizing the effects of cinnamic acid against hypertensive left ventricular hypertrophy. Cinnamic acid antagonizes the effects of ang II in inducing hypertension and left ventricular hypertrophic and fibrotic pathologies *in vivo*. Cinnamic acid directly attenuates hypertrophic phenotypes and maintains the mitochondrial function in the ang II-exposed cardiomyocytes. Moreover, cinnamic acid dampens the pro-hypertrophic activation of STAT3 and ERK1/2 triggered by not only ang II, but also IL-6, suggesting the potential of cinnamic acid against the actions of multiple pro-hypertrophic factors.

Although the pharmacological implications of cinnamic acid in pressure overload-induced hypertrophic pathologies in the heart have been demonstrated in our previous study, the impact of cinnamic acid on hypertensive left ventricular hypertrophy remains unknown. Our findings here reveal that cinnamic acid lowers blood pressure and mitigates left ventricular hypertrophic and fibrotic pathologies in ang II-infused mice *in vivo*. Cinnamic acid directly attenuates hypertrophic phenotypes and maintains the mitochondrial function in the ang II-exposed cardiomyocytes. Moreover, the mechanisms underpinning the anti-hypertrophic action of cinnamic acid in cardiomyocytes may involve its effects at suppressing the aberrant activation of STAT3 and ERK1/2 (Figure 9).

Our primary findings from this work demonstrate that cinnamic acid is pharmacologically active at mitigating ang II-mediated hypertensive left ventricular hypertrophy. We have previously shown that cinnamic acid antagonizes phenylephrine-stimulated hypertrophic responses in cardiomyocytes and attenuates left ventricular hypertrophy as well as heart failure recapitulated by TAC-induced pressure overload (Cui et al., 2023). Here, we further reveal that cinnamic acid mitigates ang II-induced hypertension and left ventricular hypertrophy. Moreover, cinnamic acid alleviates the hypertrophic responses as well as the aberrant activation of STAT3 and ERK1/2 in the ang II-stimulated cardiomyocytes, supporting that cinnamic acid is equipped with intrinsic activities to lower the level of ang II signaling in the cardiomyocytes. Of interest, the suppressive effects of cinnamic acid on STAT3 and ERK1/2 activation could be generalized to IL-6-stimulated cardiomyocytes, in which the anti-hypertrophic action of cinnamic acid is also readily detected. STAT3 is activated under a broad range of pathological conditions that lead to cardiac hypertrophy (Kunisada et al., 2000). Activated STAT3 mediates the pro-hypertrophic responses of hypertrophic stimuli, for instance, ang II and phenylephrine (Han et al., 2018; Zhang et al., 2016). In addition, by activating STAT3, IL-6 family cytokines directly induce significant hypertrophic responses in cardiomyocytes (Meléndez et al., 2010; Hirota et al., 1995; Kunisada et al., 1998). Inhibition of STAT3 leads to regression of the hypertrophic pathologies in the heart (Mir et al., 2012), whereas cardiac hypertrophic responses are noted as a result of overexpression of STAT3 in cardiomyocytes *in vivo* (Kunisada et al., 2000), supporting cell-autonomous effects of activated STAT3 in cardiac hypertrophy. In addition to STAT3, activation of ERK1/2 in cardiomyocytes occurs in response to nearly all stimuli that are known to induce hypertrophy (Bueno and Molkentin, 2002; Ueyama et al., 2000). Therefore, our previous and current findings collectively indicate that the mechanisms underpinning the anti-hypertrophic activities of cinnamic acid are different from the available target-based drug therapies. Instead of selectively blocking the action of a specific pro-hypertrophic neurohormonal factor or cytokines, for instance, phenylephrine, ang II or IL-6, cinnamic acid may act against various pro-hypertrophic stimuli in part by blocking the activation of the common signaling mediators, e.g., STAT3 and ERK1/2. This possibility is also supported by the consistent findings that support the protective effects of cinnamic on mitochondrial function in cardiomyocytes. Our previous work reveals that cinnamic acid protects against mitochondrial dysfunction and overproduction of mitochondrial ROS in the cardiomyocytes exposed to phenylephrine (Cui et al., 2023). Similar observations are also noted here in ang II-stimulated cardiomyocytes. In cardiomyocytes, ROS is actively involved in disseminating the action of pro-hypertrophic stimuli. Serving as the major source of ROS, dysfunctional mitochondria play pivotal roles in driving the pathological development of hypertrophic and failure phenotypes in the heart (Dai et al., 2011; Rosca et al., 2013; Takimoto and Kass, 2007). Therefore, the impact on mitochondrial dysfunction and mitochondrial ROS overproduction not only highlights the significance of cinnamic acid in controlling pathological cardiac hypertrophy and preventing the development of heart failure, but also support a broad action of cinnamic acid against pro-hypertrophic signaling pathways. Future investigations are required to uncover the in-depth mechanisms that contribute to the suppressive effects of cinnamic acid on the hypertrophic activation of STAT3 and ERK1/2 in cardiomyocytes. Mechanisms underlying cinnamic acid-conferred mitochondrial protection in cardiomyocytes subjected to pro-hypertrophic stimuli are also worth exploring in the future.

In addition, the findings from the ang II-infused mice and the cardiomyocytes directly exposed to ang II support the mechanisms that cinnamic acid antagonizes the vasoconstrictor activity of ang II systemically and counteracts the pro-hypertrophic action of ang II in cardiomyocytes. The newly identified blood pressure-lowering activity of cinnamic acid in ang II-infused mice corroborates the vasodilatory effects of cinnamic acid yielded by *ex vivo* vasoactivity assays (Kang et al., 2013). In the rat thoracic arteries, cinnamic acid treatment results in vasodilation in phenylephrine-precontracted aortic rings. This vasodilatory effect is abolished in the absence of the endothelium. Inhibitors against the action of endothelial nitric oxide synthase (eNOS) or guanylyl cyclase, the downstream vasodilatory target of nitric oxide in the vascular smooth muscle cells, also significantly dampen the vasodilatory activities of cinnamic acid in the aortic rings. Furthermore, in cultured endothelial cells, cinnamic acid enhances nitric oxide production presumably via promoting eNOS phosphorylation (Kang et al., 2013). Although the vasodilatory activities of cinnamic acid observed in rat aortic rings give a clue to potential anti-hypertensive activities, the *in vivo* evidence directly supporting this possibility was lacking prior to our current work. Here, by demonstrating that cinnamic acid counteracts the action of ang II in elevating blood pressure, we present new evidence that support the anti-hypertensive effects of cinnamic acid in the setting of elevated ang II signaling *in vivo*. Of note, aside from the canonical vasopressor effects via inducing the contraction of vascular smooth muscle cells, ang II also triggers endothelial dysfunction through downregulation of transient receptor potential vanilloid 4 (TRPV4), thereby sabotaging TRPV4-mediated calcium influx as well as the ensuing activation of eNOS and production of nitric oxide in endothelial cells (Kondapalli et al., 2023). Thus, aberrant ang II/TRPV4/eNOS pathway in endothelial cells is likely involved in the development of hypertension. In light of these findings and the known endothelium-dependent vasodilatory activity of cinnamic acid, it is possible that cinnamic acid exerts blood pressure-lowering effects in the ang II-infused mice via modulating eNOS/nitric oxide-dependent vasodilatory activities in endothelial cells. This possibility and in-depth mechanisms underlying the effects of cinnamic acid against ang II-mediated hypertension, for instance, whether TRPV4 and/or whether direct vasodilatory effects of cinnamic acid on endothelial cells is involved, are to be investigated in the future.

The interpretation of the current findings is limited by the lack of experiments comparing in parallel the effects of cinnamic acid with other antihypertensive drugs. Meanwhile, the experiments were done in male mice, therefore, we should be cautious generalizing the findings to female mice without validation studies. In addition, whether cinnamic acid is effective when hypertension and/or left ventricular hypertrophy is established and the long-term effects, safety and toxicity profile of cinnamic acid should be studied in the future. Moreover, the dosing regimen could have been optimized with the guidance of pharmacokinetic understanding of cinnamic acid. Nevertheless, the *in vivo* and *in vitro* evidence here suggests that cinnamic acid may serve as a lead compound to be optimized and developed as new drugs to mitigate hypertensive left ventricular hypertrophy. Meanwhile, based on the evidence from the current work as well as our previous findings that support the antagonism of cinnamic acid to phenylephrine-induced cardiomyocyte hypertrophy *in vitro* and TAC-induced left ventricular hypertrophy *in vivo* (Cui et al., 2023), it is reasonable to propose that cinnamic acid is equipped with multi-targeted effects against cardiomyocyte hypertrophy. Thus, the potential synergy and interaction between cinnamic acid and other antihypertensive drugs should also be assessed in the future to address the possibility of cinnamic acid as an adjunct therapy for left ventricular hypertrophy.

In conclusion, our work here demonstrates for the first time that cinnamic acid is effective at mitigating hypertensive left ventricular hypertrophy. We report new pharmacological activities of cinnamic acid at counteracting the actions of ang II in inducing hypertension and left ventricular hypertrophic responses *in vivo*. Furthermore, we identify new mechanisms of action of cinnamic acid against the pro-hypertrophic activation of STAT3 and ERK1/2 in both ang II and IL-6-stimulated cardiomyocytes, which may in part underpin the beneficial effects of cinnamic acid in controlling the development of hypertensive left ventricular hypertrophy.

## Data Availability

The raw data supporting the conclusions of this article will be made available by the authors, without undue reservation.

## References

[B1] Anupama N. Preetha Rani M. R. Shyni G. L. Raghu K. G. (2018). Glucotoxicity results in apoptosis in H9c2 cells via alteration in redox homeostasis linked mitochondrial dynamics and polyol pathway and possible reversal with cinnamic acid. Toxicol Vitro 53, 178–192. 10.1016/j.tiv.2018.08.010 30144576

[B2] Aronow W. S. (2017). Hypertension and left ventricular hypertrophy. Ann. Transl. Med. 5, 310. 10.21037/atm.2017.06.14 28856150 PMC5555990

[B3] Atanasov A. G. Zotchev S. B. Dirsch V. M. Supuran C. T. (2021). Natural products in drug discovery: advances and opportunities. Nat. Rev. Drug Discov. 20, 200–216. 10.1038/s41573-020-00114-z 33510482 PMC7841765

[B4] Blaustein M. P. (2017). How does pressure overload cause cardiac hypertrophy and dysfunction? High-ouabain affinity cardiac Na(+) pumps are crucial. Am. J. Physiol. Heart Circ. Physiol. 313, H919–H930. 10.1152/ajpheart.00131.2017 28733446 PMC5792198

[B5] Bueno O. F. Molkentin J. D. (2002). Involvement of extracellular signal-regulated kinases 1/2 in cardiac hypertrophy and cell death. Circ. Res. 91, 776–781. 10.1161/01.res.0000038488.38975.1a 12411391

[B6] Chen J. Zhang Y. Wang Y. Jiang P. Zhou G. Li Z. (2021). Potential mechanisms of Guizhi decoction against hypertension based on network pharmacology and Dahl salt-sensitive rat model. Chin. Med. 16, 34. 10.1186/s13020-021-00446-x 33906674 PMC8077739

[B7] Cui Y. Wang P. Li M. Wang Y. Tang X. Cui J. (2023). Cinnamic acid mitigates left ventricular hypertrophy and heart failure in part through modulating FTO-dependent N(6)-methyladenosine RNA modification in cardiomyocytes. Biomed. Pharmacother. 165, 115168. 10.1016/j.biopha.2023.115168 37453198

[B8] Cuspidi C. Sala C. Negri F. Mancia G. Morganti A. Italian Society of Hypertension (2012). Prevalence of left-ventricular hypertrophy in hypertension: an updated review of echocardiographic studies. J. Hum. Hypertens. 26, 343–349. 10.1038/jhh.2011.104 22113443

[B9] Dai D. F. Johnson S. C. Villarin J. J. Chin M. T. Nieves-Cintrón M. Chen T. (2011). Mitochondrial oxidative stress mediates angiotensin II-induced cardiac hypertrophy and Galphaq overexpression-induced heart failure. Circ. Res. 108, 837–846. 10.1161/CIRCRESAHA.110.232306 21311045 PMC3785241

[B10] Ding L. Jia C. Zhang Y. Wang W. Zhu W. Chen Y. (2019). Baicalin relaxes vascular smooth muscle and lowers blood pressure in spontaneously hypertensive rats. Biomed. Pharmacother. 111, 325–330. 10.1016/j.biopha.2018.12.086 30590320

[B11] Han J. Ye S. Zou C. Chen T. Wang J. Li J. (2018). Angiotensin II causes biphasic STAT3 activation through TLR4 to initiate cardiac remodeling. Hypertension 72, 1301–1311. 10.1161/HYPERTENSIONAHA.118.11860 30571233

[B12] Hirota H. Yoshida K. Kishimoto T. Taga T. (1995). Continuous activation of gp130, a signal-transducing receptor component for interleukin 6-related cytokines, causes myocardial hypertrophy in mice. Proc. Natl. Acad. Sci. U. S. A. 92, 4862–4866. 10.1073/pnas.92.11.4862 7539136 PMC41807

[B13] Hoskins J. A. (1984). The occurrence, metabolism and toxicity of cinnamic acid and related compounds. J. Appl. Toxicol. 4, 283–292. 10.1002/jat.2550040602 6394637

[B14] Kang Y. H. Kang J. S. Shin H. M. (2013). Vasodilatory effects of cinnamic acid via the nitric oxide-cGMP-PKG pathway in rat thoracic aorta. Phytother. Res. 27, 205–211. 10.1002/ptr.4708 22517576

[B15] Koczurkiewicz-Adamczyk P. Klaś K. Gunia-Krzyżak A. Piska K. Andrysiak K. Stępniewski J. (2021). Cinnamic acid derivatives as cardioprotective agents against oxidative and structural damage induced by doxorubicin. Int. J. Mol. Sci. 22, 6217. 10.3390/ijms22126217 34207549 PMC8227863

[B16] Kondapalli N. B. Katari V. Dalal K. Paruchuri S. Thodeti C. K. (2023). Angiotensin II induces endothelial dysfunction and vascular remodeling by downregulating TRPV4 channels. J. Mol. Cell Cardiol. Plus 6, 100055. 10.1016/j.jmccpl.2023.100055 38333200 PMC10852140

[B17] Kunisada K. Negoro S. Tone E. Funamoto M. Osugi T. Yamada S. (2000). Signal transducer and activator of transcription 3 in the heart transduces not only a hypertrophic signal but a protective signal against doxorubicin-induced cardiomyopathy. Proc. Natl. Acad. Sci. U. S. A. 97, 315–319. 10.1073/pnas.97.1.315 10618415 PMC26660

[B18] Kunisada K. Tone E. Fujio Y. Matsui H. Yamauchi-Takihara K. Kishimoto T. (1998). Activation of gp130 transduces hypertrophic signals via STAT3 in cardiac myocytes. Circulation 98, 346–352. 10.1161/01.cir.98.4.346 9711940

[B19] Kurisu S. Ozono R. Oshima T. Kambe M. Ishida T. Sugino H. (2003). Cardiac angiotensin II type 2 receptor activates the kinin/NO system and inhibits fibrosis. Hypertension 41, 99–107. 10.1161/01.hyp.0000050101.90932.14 12511537

[B20] Luan F. Rao Z. Peng L. Lei Z. Zeng J. Peng X. (2022). Cinnamic acid preserves against myocardial ischemia/reperfusion injury via suppression of NLRP3/Caspase-1/GSDMD signaling pathway. Phytomedicine 100, 154047. 10.1016/j.phymed.2022.154047 35320770

[B21] Meléndez G. C. McLarty J. L. Levick S. P. Du Y. Janicki J. S. Brower G. L. (2010). Interleukin 6 mediates myocardial fibrosis, concentric hypertrophy, and diastolic dysfunction in rats. Hypertension 56, 225–231. 10.1161/HYPERTENSIONAHA.109.148635 20606113 PMC2921860

[B22] Messerli F. H. Schmieder R. (1986). Left ventricular hypertrophy. A cardiovascular risk factor in essential hypertension. Drugs 31 (Suppl. 4), 192–201. 10.2165/00003495-198600314-00023 2942387

[B23] Mir S. A. Chatterjee A. Mitra A. Pathak K. Mahata S. K. Sarkar S. (2012). Inhibition of signal transducer and activator of transcription 3 (STAT3) attenuates interleukin-6 (IL-6)-induced collagen synthesis and resultant hypertrophy in rat heart. J. Biol. Chem. 287, 2666–2677. 10.1074/jbc.M111.246173 22157761 PMC3268425

[B24] Mohammadabadi T. Jain R. (2024). Cinnamon: a nutraceutical supplement for the cardiovascular system. Arch. Med. Sci. Atheroscler. Dis. 9, e72–e82. 10.5114/amsad/184245 38846056 PMC11155465

[B25] Nair A. Preetha Rani M. R. Salin Raj P. Ranjit S. Rajankutty K. Raghu K. G. (2022). Cinnamic acid is beneficial to diabetic cardiomyopathy via its cardioprotective, anti-inflammatory, anti-dyslipidemia, and antidiabetic properties. J. Biochem. Mol. Toxicol. 36, e23215. 10.1002/jbt.23215 36117386

[B26] Osterholt M. Nguyen T. D. Schwarzer M. Doenst T. (2013). Alterations in mitochondrial function in cardiac hypertrophy and heart failure. Heart Fail Rev. 18, 645–656. 10.1007/s10741-012-9346-7 22968404

[B27] Qu J. Wang J. Zheng B. Jiang X. Liu J. Chen J. (2023). Exploring the effects and mechanisms of Guizhigancao Decoction on heart failure using an integrated approach based on experimental support and network pharmacology strategy. J. Tradit. Complement. Med. 13, 454–464. 10.1016/j.jtcme.2023.03.010 37693095 PMC10491989

[B28] Roger V. L. (2021). Epidemiology of heart failure: a contemporary perspective. Circ. Res. 128, 1421–1434. 10.1161/CIRCRESAHA.121.318172 33983838

[B29] Rosca M. G. Tandler B. Hoppel C. L. (2013). Mitochondria in cardiac hypertrophy and heart failure. J. Mol. Cell Cardiol. 55, 31–41. 10.1016/j.yjmcc.2012.09.002 22982369 PMC3805050

[B30] Schiattarella G. G. Hill J. A. (2015). Inhibition of hypertrophy is a good therapeutic strategy in ventricular pressure overload. Circulation 131, 1435–1447. 10.1161/CIRCULATIONAHA.115.013894 25901069 PMC4408778

[B31] Schnee J. M. Hsueh W. A. (2000). Angiotensin II, adhesion, and cardiac fibrosis. Cardiovasc Res. 46, 264–268. 10.1016/s0008-6363(00)00044-4 10773230

[B32] Song F. Li H. Sun J. Wang S. (2013). Protective effects of cinnamic acid and cinnamic aldehyde on isoproterenol-induced acute myocardial ischemia in rats. J. Ethnopharmacol. 150, 125–130. 10.1016/j.jep.2013.08.019 24001892

[B33] Takimoto E. Kass D. A. (2007). Role of oxidative stress in cardiac hypertrophy and remodeling. Hypertension 49, 241–248. 10.1161/01.HYP.0000254415.31362.a7 17190878

[B34] Ueyama T. Kawashima S. Sakoda T. Rikitake Y. Ishida T. Kawai M. (2000). Requirement of activation of the extracellular signal-regulated kinase cascade in myocardial cell hypertrophy. J. Mol. Cell Cardiol. 32, 947–960. 10.1006/jmcc.2000.1135 10888249

[B35] Wang L. Zhang Y. L. Lin Q. Y. Liu Y. Guan X. M. Ma X. L. (2018). CXCL1-CXCR2 axis mediates angiotensin II-induced cardiac hypertrophy and remodelling through regulation of monocyte infiltration. Eur. Heart J. 39, 1818–1831. 10.1093/eurheartj/ehy085 29514257

[B36] Wang S. Cui Y. Xiong M. Li M. Wang P. Cui J. (2021). Dual activity of ginsenoside Rb1 in hypertrophic cardiomyocytes and activated macrophages: implications for the therapeutic intervention of cardiac hypertrophy. J. Inflamm. Res. 14, 1789–1806. 10.2147/JIR.S310633 33981156 PMC8108398

[B37] Wu D. Ding L. Tang X. Wang W. Chen Y. Zhang T. (2019). Baicalin protects against hypertension-associated intestinal barrier impairment in part through enhanced microbial production of short-chain fatty acids. Front. Pharmacol. 10, 1271. 10.3389/fphar.2019.01271 31719823 PMC6826474

[B38] Zablocki D. Sadoshima J. (2013). Solving the cardiac hypertrophy riddle: the angiotensin II-mechanical stress connection. Circ. Res. 113, 1192–1195. 10.1161/CIRCRESAHA.113.302501 24201113

[B39] Zhang X. Li W. Shen P. Feng X. Yue Z. Lu J. (2016). STAT3 suppression is involved in the protective effect of SIRT6 against cardiomyocyte hypertrophy. J. Cardiovasc Pharmacol. 68, 204–214. 10.1097/FJC.0000000000000404 27124607

